# Mobile and traditional cognitive behavioral therapy programs for generalized anxiety disorder: A cost-effectiveness analysis

**DOI:** 10.1371/journal.pone.0190554

**Published:** 2018-01-04

**Authors:** Shefali Kumar, Megan Jones Bell, Jessie L. Juusola

**Affiliations:** 1 Evidation Health, San Mateo, California, United States of America; 2 Department of Psychiatry and Behavioral Sciences, Stanford University School of Medicine, Stanford, California, United States of America; Stellenbosch University, SOUTH AFRICA

## Abstract

**Background:**

Generalized anxiety disorder (GAD) is a debilitating mental health illness that affects approximately 3.1% of U.S. adults and can be treated with cognitive behavioral therapy (CBT). With the emergence of digital health technologies, mobile CBT may be a cost-effective way to deliver care. We developed an analysis framework to quantify the cost-effectiveness of internet-based CBT for individuals with GAD. As a case study, we examined the potential value of a new mobile-delivered CBT program for GAD.

**Methods:**

We developed a Markov model of GAD health states combined with a detailed economic analysis for a cohort of adults with GAD in the U.S. In our case study, we used pilot program efficacy data to evaluate a mobile CBT program as either prevention or treatment only and compared the strategies to traditional CBT and no CBT. Traditional CBT efficacy was estimated from clinical trial results. We calculated discounted incremental costs and quality-adjusted life-years (QALYs) over the cohort lifetime.

**Case study results:**

In the base case, for a cohort of 100,000 persons with GAD, we found that mobile CBT is cost-saving. It leads to a gain of 34,108 QALYs and 81,492 QALYs and a cost reduction of $2.23 billion and $4.54 billion when compared to traditional CBT and no CBT respectively. Results were insensitive to most model inputs and mobile CBT remained cost-saving in almost all scenarios.

**Limitations:**

The case study was conducted for illustrative purposes and used mobile CBT efficacy data from a small pilot program; the analysis should be re-conducted once robust efficacy data is available. The model was limited in its ability to measure the effectiveness of CBT in combination with pharmacotherapy.

**Conclusions:**

Mobile CBT may lead to improved health outcomes at lower costs than traditional CBT or no intervention and may be effective as either prevention or treatment.

## Introduction

Approximately 3.1% of the adult population in the United States (6.8 million adults) is affected by generalized anxiety disorder (GAD), a chronic and debilitating mental health illness that is characterized by low spontaneous remission rates and frequent relapse after remission [1–3]. Individuals with GAD experience persistent worry and anxiety, which often interferes with their ability to perform routine activities and function normally in social settings and the workplace [4]. Studies have shown that GAD is associated with a significant societal burden, including reduced economic productivity and increased healthcare utilization [5, 6]. Up to 60% of individuals with anxiety also experience comorbid mental health illnesses and addictions, particularly major depressive disorder (MDD) and substance abuse, as well as social anxiety disorder and post-traumatic stress disorder [4, 5, 7–9]. This is associated with even worse disability, diminished functioning and impairment [4].

While GAD can be treated with pharmacotherapy, studies have demonstrated that cognitive behavioral therapy (CBT) is also an effective treatment method for GAD [10]. However, many individuals with GAD in the U.S. do not receive adequate or even any amount of treatment [11, 12]. One analysis using data from the National Comorbidity Survey found that only 20% of individuals with GAD visited a healthcare specialist (e.g., psychologist, psychiatrist) in a given year [12]. This treatment gap is partially due to structural and financial barriers that prevent access to healthcare specialists and traditional face-to-face CBT [13]. Internet-based interventions may provide an effective but more widely-accessible and cost-effective alternative to traditional CBT [14, 15], and may also offer greater opportunity to utilize CBT as prevention, where research indicates it may also be effective [16].

A recent systematic review showed that internet-based CBT programs were cost-effective when compared to status quo for individuals with broad anxiety disorders [17]. However, most cost-effectiveness analyses and economic evaluations that examine CBT programs have focused on multiple anxiety disorders (e.g., GAD, panic disorder and obsessive compulsive disorder); for models and analyses that do specifically look only at a population with GAD, the intervention(s) being examined are often various pharmacotherapy options, not CBT programs [17]. In addition, despite the value of conducting a cost-effectiveness analysis from both the payer and societal perspective [18], the majority of economic models and evaluations that examine anxiety interventions do not take the societal perspective [17]. Overall, there is limited research on the cost-effectiveness of internet-based CBT programs specifically for GAD in the U.S., as compared to traditional CBT or no CBT, from both the payer and the societal perspective [6, 17].

In this study, we aimed to develop a model and a framework for analysis that would help researchers quantify the cost-effectiveness of internet-based CBT for individuals with GAD in the U.S. from both a payer and societal perspective. As a case study and as an application of this model, we used this framework to examine the potential value of a new mobile-delivered, guided self-help CBT program for GAD (Thrive Network, Inc. [DBA, Lantern], San Francisco, CA), which we refer to as mobile CBT [19, 20]. We used initial clinical effectiveness data for this mobile CBT program to estimate the program’s potential cost-effectiveness as compared to traditional or no CBT.

## Methods

### Overview and model structure

We designed and built a computer-simulated Markov model of GAD progression and treatment to assess the effectiveness and cost-effectiveness of GAD treatment and prevention programs for a cohort of 100,000 adults with GAD in the U.S. We calibrated the model to published literature reporting the cost of healthcare utilization for GAD [6, 9, 21], and estimated the quality-adjusted life-years (QALYs) gained and the costs of various GAD prevention and treatment strategies over the cohort lifetime. We ran the model with a societal perspective as well as a payer perspective, and discounted costs and QALYs at 3% annually [18]. We implemented the model in TreeAge Pro 2016 (TreeAge Software, Williamstown, Massachusetts). Key model parameters are presented in Table 1.

**Table 1:** Summary of key model parameters.

Parameter	Value	Range	Source
**General and Demographic Parameters**			
Time horizon, months	3	-	Assumed
Cohort size	100,000	-	Assumed
Cohort starting age, years	38	-	Expert opinion [22]
3-month mortality rate	.0003–.0711	-	[23]
Proportion of population on medication, %	58.6	0–100	[24]
Starting disease state: base-case, %			
Mild	32.3	-	[1]
Moderate	44.6	-	[1]
Severe	23.1	-	[1]
Starting disease state: prevention only, %			
Mild	100	-	Assumed
Moderate	0	-	Assumed
Severe	0	-	Assumed
Starting disease state: treatment only, %			
Mild	0	-	Assumption
Moderate	65.9	-	Calculated [1]
Severe	34.1	-	Calculated [1]
**Health State Utilities**			
No anxiety	0.80	-	[25]
Mild anxiety	0.72	-	[25]
Moderate anxiety	0.68	-	[25]
Severe anxiety	0.64	-	[25]
Moderate anxiety with comorbidities	0.56	-	[25]
Severe anxiety with comorbidities	0.48	-	[25]
Dead	0	-	Assumed
**Cost Parameters, $**^a^			
Annual baseline non-GAD healthcare costs	946–5,154	-	[26]
Cost of 3-month course of CBT^b^	1,200	960–1,440	Expert opinion [27]
Cost of mobile CBT program	150	120–180	Expert opinion
Pharmaceutical treatment^c^	105	84–126	[24]
Physician visit	143	115–172	[28]
ER visits	782	625–938	[28]
Hospitalization^d^	8,986	7,189–10,739	[28]
Disability day (productivity impact)	206	165–247	[29]

CBT: Cognitive behavior therapy; ER: emergency room

^a^ In 2016 U.S. dollars

^b^ Cost of 3-month CBT program was based on a conservative average of 10 sessions (assuming not all patients complete the recommended 12–20 CBT sessions) at $120 per session

^c^ Cost of pharmaceutical therapy was based on the average 3-month cost of anxiety-related pharmaceutical therapy using U.S. claims data

^d^ Cost of hospitalization was based on the average cost of an anxiety-related hospitalization

### Structure of GAD model

**Fig 1:**
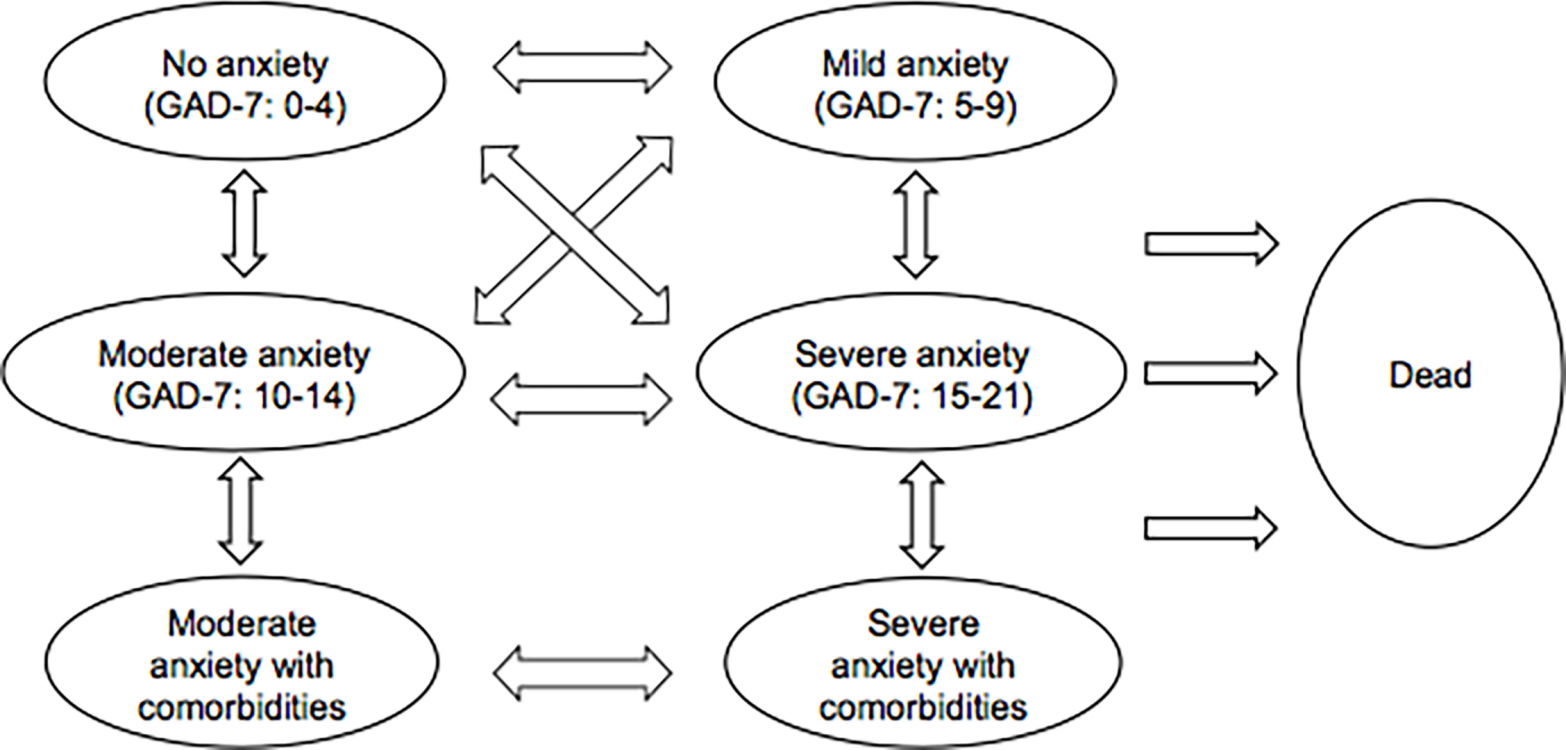
Markov model structure. This model includes 7 health states where persons can move between states at 3-month intervals. In the base case, all persons begin in the “mild anxiety,” “moderate anxiety” or “severe anxiety” states. In the prevention only case, all persons begin in the “mild anxiety” state. In the treatment only case, all persons begin in the “moderate anxiety” or “severe anxiety” state. Every cycle, persons could transition to a healthier GAD state, progress to a more severe GAD state (e.g., relapse), remain in the same state, or die.

Our Markov model includes 7 health states (Fig 1) with a 3-month cycle length [30, 31]. The 4 anxiety states are based on the cut-off scores from the Generalized Anxiety Disorder-7 (GAD-7) scale, with the first state being “no anxiety,” defined as GAD-7 of 0 to 4 [32]. In the base case analysis, persons began in the “mild anxiety,” “moderate anxiety” or “severe anxiety” states. Every cycle, persons could transition to a healthier GAD state, progress to a more severe GAD state (e.g., relapse), stay at the same state, or die. In order to measure the full clinical and economic burden of GAD, we included a “moderate anxiety with comorbidities” state and “severe anxiety with comorbidities” state in our model, where comorbidities were defined as MDD and/or substance abuse. These comorbidities occur often with clinically diagnosed GAD and can greatly impact health-related costs and QALYs [5, 8, 9]. Persons could only progress to these states after the first cycle. Transition rates between states were estimated from the published literature (S1 Table). We adhered to guidelines for best practice procedures for economic modeling in designing and parameterizing the model [18].

Pharmaceutical drugs are often used to treat GAD alone or in combination with CBT [24]. In our model, we assumed that 58.6% of the cohort was consistently on some form of pharmacotherapy, based on published literature [24]. This proportion remained constant for all strategies examined, and did not change as an individual moved between states, due to a lack of data breaking out GAD progression by whether a person is on medication. Hence, pharmacotherapy was only factored into cost calculations and did not impact transition rates.

### Populations targeted

Persons with no or mild anxiety (GAD-7 score of 0–9) usually do not have clinically diagnosable anxiety and are not treated [32, 33]. Persons with moderate or severe anxiety (GAD score of 10–21) likely have clinically diagnosable GAD [32] and are recommended to receive treatment [33]. In order to evaluate an internet-based CBT program as treatment, prevention or both, we examined 3 scenarios for analysis. In the base case, the program was used as both prevention and treatment, and the starting cohort was distributed amongst the “mild anxiety,” “moderate anxiety” and “severe anxiety” states (Table 1). In the prevention-only scenario, the entire cohort started in the “mild anxiety” state with CBT as a preventive intervention. In the treatment-only scenario, the entire starting cohort was either in the “moderate anxiety” or “severe anxiety” states.

### GAD prevention and treatment strategies

We considered two options as comparators to internet-based CBT programs for this analysis: traditional face-to-face CBT programs and status quo. Per cost-effectiveness analysis guidelines, considering multiple comparators is beneficial, and comparators should be relevant alternatives to the intervention [18]. Traditional face-to-face CBT is a current alternative to internet-based CBT programs and can be used for GAD prevention and treatment. The status quo strategy assumed that patients did not receive CBT, but potentially received other forms of treatment such as pharmacotherapy.

We assumed that GAD patients receiving either the internet-based or traditional face-to-face CBT program would begin immediately and would participate in the program for 3 months, which is the equivalent to approximately 12–20 CBT sessions as suggested by the Anxiety and Depression American Association treatment guidelines [33].

For the strategy of traditional face-to-face CBT programs, we assumed standard program characteristics based on available literature. A CBT session is a one-on-one meeting between a patient and a trained therapist that takes place in an in-office setting. Sessions are typically one hour long and cover relaxation techniques, psychoeducation, and problem-solving techniques [33].

Traditional face-to-face CBT efficacy data came from a published Cochrane review on GAD therapy effectiveness. Results from 6 studies examining CBT as treatment for GAD showed that approximately 42% of study participants clinically responded to CBT (i.e., moved to a lower GAD state) [34]. This clinical response rate was applied to the various anxiety states to match the structure of the state-specific efficacy data of the Markov model (S1 Table). The review did not provide information on completion rates, so we assumed that the clinical response rate reported from these studies was based on an intent-to-treat population, and therefore assumed that program completion rates were already factored into efficacy data [34].

The model included a time-based linear function to capture both the traditional face-to-face CBT and internet-based CBT strategies’ waning effect over time [35]. The interventions’ effect was fully sustained for the first year, or the first 4 model cycles [35]. This included the first 3 months when persons were receiving the intervention, and the following 9 months after they received the intervention. After this first year, the effectiveness of the intervention gradually decreases over the lifetime of the cohort until the state transition rates are the same as for the status quo at the end of the model timeframe.

### Case study: Mobile CBT

To illustrate the potential impact of internet-based CBT programs using our model and proposed analysis, we used pilot program efficacy data for a particular new mobile CBT program. This mobile CBT program for GAD is based on the work of Newman et al. and allows individuals to complete interactive CBT learnings and techniques to help them manage their anxiety and receive individualized support from a coach over a 3-month program [36, 37]. The mobile CBT program is comprised of 8 self-guided CBT-based modules that participants complete, including modules on relaxation, mindfulness, cognitive reframing and behavior change. These modules include a combination of education and practice, including daily audio-guided or self-guided interactive and personalized CBT techniques to help them manage their anxiety. A trained coach, with a health and wellness coaching or psychology background, provides support and motivational enhancement via asynchronous in-app messages. Participants can access the program on a smartphone application. Mobile CBT efficacy data came from a pilot program conducted at a large national employer group of approximately 10,000 employees. Eligible employees were offered access to the mobile CBT program, and 410 enrolled. Of those who enrolled, 89 completed baseline and month 3 GAD-7 questionnaires, and provided consent. Persons provided consent, via the smartphone application, for their data to be used for research purposes prior to participation in the program. The mobile CBT program is HIPAA compliant and de-identified data was provided by the program developer to the researchers using an encrypted, secure data transfer. Given that this initiative was designed as a program evaluation, no institutional review board (IRB) approval was obtained. Data analysis on a sample of 89 individuals from the pilot program was conducted independently by the Evidation Health researchers.

Before enrolling in the mobile CBT program, persons completed a baseline GAD-7 screen. At the end of the 3-month program, persons completed another GAD-7 test [32]. Overall, 70% of participants clinically responded to CBT (i.e., moved to a lower GAD state); state-specific mobile CBT program efficacy was calculated directly from pilot program data (S1 Table). Effect sizes seen in this pilot program were comparable to efficacy demonstrated from other larger, robust studies examining internet-based CBT programs [15, 38]. This mobile CBT efficacy data replaced the transition rates for individuals in the “mild anxiety,” “moderate anxiety,” or “severe anxiety” states. Given the structure and design of the pilot, it can be assumed that the majority of the participants who completed the 3-month GAD-7 test also completed the mobile CBT program, and thus program completion rates were implicitly factored into efficacy data.

### Health outcomes and costs

We simulated the population over time and calculated discounted costs, discounted QALYs and cumulative (undiscounted) life years with and without anxiety for each scenario. We estimated quality of life for each health state and adjusted the utilities based on the average age of the modeled population. Quality-of-life data came from a study examining health-related quality of life and utilities for persons with GAD [25]. We used the results of a regression analysis from that study to predict state-specific quality of life, reported using the SF-6D score (Table 1). Age-based mortality rates were used to transition persons to the dead state [23]. Persons in the comorbidities states experienced a higher rate of mortality due to increased suicide risk [39].

For the societal perspective, we included costs associated with baseline medical care in each state, cost of the interventions, pharmaceutical costs, and costs associated with disability days to calculate total health-related costs. For the payer perspective, we did not include disability day costs. Baseline medical costs and pharmaceutical costs were estimated from the published literature. Physician office visits, emergency room visits and hospitalization costs were estimated using data from the 2013 Medical Expenditure Panel (S2 Table) [28]. Disability days costs were calculated by multiplying the number of disability days by the average daily wage in the U.S., as reported by the U.S. Bureau of Labor Statistics [29]. All costs were adjusted to 2016 U.S. dollars using the overall Consumer Price Index [40].

### Sensitivity analyses

We conducted a number of sensitivity analyses to characterize the robustness of the model and examine the findings of our case study. Our sensitivity analyses included varying all healthcare costs and utilization inputs, as well as the cost of disability days, to measure the impact of these inputs on overall model results. We also examined how varying mobile CBT program effectiveness, and the sustained effectiveness of mobile and traditional CBT programs, impacted model results. We also varied the probability of death due to suicide in our sensitivity analyses.

## Case study results

### Societal perspective

In all scenarios examined in the case study, the mobile CBT program for GAD led to improved health outcomes and lower costs than traditional CBT or the status quo. In the base case, for a cohort of 100,000 persons with mild, moderate, or severe anxiety, we found that mobile CBT led to a gain of 34,108 QALYs when compared to traditional CBT and 81,492 QALYs when compared to the status quo (Table 2). Under the mobile CBT strategy, the cohort resulted in 191,000 and 834,000 more cumulative (undiscounted) life years with no anxiety compared to traditional CBT and the status quo respectively (Table 3). The cohort also spent 127,000 and 253,000 fewer cumulative life years in the clinically diagnosable GAD states when compared to traditional CBT and the status quo respectively.

**Table 2:** Total reduction in costs and improvement in QALYs.

	Mobile CBT Compared to Traditional CBT^a^		Mobile CBT Compared to Status Quo^a^	
	Reduction in Costs, million $	Improvement in QALYs	Reduction in Costs, million $	Improvement in QALYs
**Societal Perspective**				
Base case	2,234	34,108	4,545	81,492
Prevention only	1,926	28,959	4,152	76,568
Treatment only	2,381	36,564	4,733	83,841
**Payer Perspective**				
Base case	339	34,108	605	81,492
Prevention only	297	28,959	553	76,568
Treatment only	360	36,564	630	83,841

CBT: cognitive behavioral therapy; QALY: quality-adjusted life years

^a^ Costs and QALYs are net present values (3% annual discount rate) over the cohort lifetime

**Table 3:** Summary of cumulative per-person life-years spent in each state.

	Mobile CBT	Traditional CBT	Status quo
**Base Case**			
No anxiety	27.91	26.00	19.57
Mild anxiety	7.09	6.27	11.42
Moderate anxiety	1.57	2.18	2.51
Severe anxiety	2.77	4.01	4.46
Moderate anxiety with comorbidities	0.37	0.56	0.66
Severe anxiety with comorbidities	1.06	1.74	2.12
**Prevention Only**			
No anxiety	28.01	26.35	19.52
Mild anxiety	7.25	6.45	12.25
Moderate anxiety	1.44	1.94	2.22
Severe anxiety	2.72	3.87	4.22
Moderate anxiety with comorbidities	0.35	0.51	0.60
Severe anxiety with comorbidities	1.02	1.65	1.96
**Treatment Only**			
No anxiety	27.86	25.83	19.60
Mild anxiety	7.02	6.19	11.03
Moderate anxiety	1.63	2.3	2.65
Severe anxiety	2.80	4.07	4.58
Moderate anxiety with comorbidities	0.38	0.58	0.69
Severe anxiety with comorbidities	1.08	1.79	2.20

CBT: cognitive behavioral therapy

From our analysis in this case study, we estimated that with mobile CBT, the 100,000 person cohort costs approximately $36.5 billion over their lifetime compared to $38.7 billion and $41.0 billion with traditional CBT and status quo (Table 4). Mobile CBT reduced overall costs by approximately $2.23 billion when compared to traditional CBT (Table 2). Approximately 85% of these savings ($1.89 billion) came from reduced disability days and 15% ($340 million) came from healthcare costs (S3 Table). Mobile CBT reduced overall costs by approximately $4.55 billion when compared to the status quo (Table 2). Approximately 87% of these savings ($3.94 billion) came from reduced disability days and the remaining 13% of savings ($604 million) came from healthcare utilization costs (S3 Table). Intervention costs were higher in the mobile CBT scenario than status quo but were outweighed by the savings from disability days and healthcare utilization.

**Table 4:** Total costs for CBT interventions and status quo.

	Mobile CBT, billion $^a^	Traditional CBT, billion $ ^a^	Status quo, billion $ ^a^
**Societal Perspective**			
Base case	36.5	38.7	41.0
Prevention only	36.3	38.2	40.4
Treatment only	36.6	38.9	41.3
**Payer Perspective**			
Base case	24.9	25.3	25.5
Prevention only	24.9	25.2	25.4
Treatment only	24.9	25.3	25.6

CBT: cognitive behavioral therapy

^a^ Costs and QALYs are net present values (3% annual discount rate) over the cohort lifetime

In the prevention-only scenario, mobile CBT resulted in fewer QALYs gained and costs saved than in the base case scenario but was still cost-saving. Mobile CBT increased QALYs by 28,959 and 76,568 and reduced overall costs by $1.93 billion and $4.15 billion when compared to traditional CBT and the status quo respectively. In contrast, in the treatment-only scenario, mobile CBT had a greater impact than in the base case. Mobile CBT increased QALYs by 36,564 and 83,841 and reduced overall costs by $2.38 billion and $4.73 billion when compared to CBT and the status quo respectively (Table 2).

### Payer perspective

From a payer perspective, the mobile CBT program for GAD also resulted in lower costs compared to traditional CBT or the status quo. In the base case, we estimated that mobile CBT reduces overall costs by approximately $339 million when compared to traditional CBT (Table 2). Approximately 43% ($146 million) of these savings came from reduced physician visits, 41% ($139 million) came from reduced hospitalizations, and 3% ($9.1 million) came from reduced ER visits (S3 Table). Mobile CBT reduced overall costs by approximately $605 million when compared to status quo (Table 2). The majority of these savings came from reduced physician visits ($327 million) and reduced hospitalizations ($280 million) (S3 Table).

From a payer perspective in the prevention-only scenario, mobile CBT also resulted in fewer costs saved than in the base case scenario. When compared to traditional CBT and status quo, mobile CBT reduced overall costs by $297 million and $553 million respectively. In contrast, in the treatment-only scenario, mobile CBT had a greater impact than in the base case. When compared to CBT and the status quo, mobile CBT reduced overall costs by approximately $360 million and $630 million respectively (Table 2).

### Sensitivity analysis

In sensitivity analysis, we found that results were insensitive to most model inputs and that mobile CBT remained cost-saving compared to traditional CBT and the status quo in almost all scenarios tested. The model parameters adjusted for the sensitivity analysis are summarized in Table 1. We first looked at the effectiveness of the CBT programs. When varying the number of years the mobile and traditional CBT programs’ effectiveness was sustained, mobile CBT was still cost-saving compared to traditional CBT and the status quo. When the effectiveness of both CBT programs gradually decreased over 2 years until it reached the status quo transition rates, versus over the cohort lifetime in the base case, then mobile CBT reduced overall costs by approximately $435 million and $750 million and increased QALYs by 6,297 and 14,180 respectively. Additionally, we varied the clinical response rate of traditional CBT to measure the threshold at which traditional CBT becomes the preferred treatment. When the clinical response rate of traditional CBT was increased to 76%, and the effectiveness of mobile CBT did not change, then traditional CBT becomes cost-effective as compared to mobile CBT. Lastly, we decreased the effectiveness of the mobile CBT program by 20%. We found that mobile CBT was still cost-saving compared to traditional CBT and the status quo, and reduced overall costs by $1.50 billion and $3.81 billion and increased QALYs by 21,116 and 68,500 respectively.

In the base case from the societal perspective, reduction in disability day cost made up much of the savings from mobile CBT. Taking a more conservative approach to measuring cost of disability days and assuming that for a given disability day, an individual is 80% productive (decreasing the costs associated with each disability day by 80%), we then found that mobile CBT still reduced costs compared to traditional CBT (reduction of $719 million) and the status quo (reduction of $1.39 billion).

Lastly, we examined the impact of suicide risk on costs and QALYs. We found that results were insensitive to moderate variations in probability of death due to suicide. In the base case, increased suicide risk was only applied to the “moderate with comorbidities” and “severe with comorbidities” model states. In sensitivity analysis, we applied the same suicide risk used in the “moderate anxiety with comorbidities” and “severe anxiety with comorbidities” states to the “moderate anxiety” and “severe anxiety” states respectively. With these adjustments, we found that mobile CBT still reduced costs and was additive to QALYs. When compared to traditional CBT and status quo, mobile CBT reduced overall costs by approximately $2.20 billion and $4.09 billion and increased QALYs by 35,000 and 83,000 respectively.

## Discussion

We developed a model and framework that can help researchers measure the cost-effectiveness of internet-based CBT for individuals with GAD. Our model is specific to the U.S., and our suggested analysis framework calculates cost-effectiveness from both a payer and a societal perspective.

In our case study analysis, we found that in all three intervention scenarios, the mobile CBT program for GAD would increase QALYs and reduce health-related costs both from a societal and a payer perspective when compared to traditional CBT and the status quo. Using the model, we estimated that, for a cohort of 100,000 adults with GAD, the societal burden associated with GAD could be reduced by $2.23 billion if mobile CBT were to be used instead of traditional CBT and by $4.55 billion if mobile CBT is compared to the status quo of no CBT. Much of this savings came from a reduction in disability days (85% and 87% respectively), but results held from a payer perspective as well, when disability days were excluded. Total savings from mobile CBT were $339 million and $605 million respectively in that case. In this analysis, the mobile CBT was cost-saving primarily due to the better clinical response rate and lower program costs for mobile CBT than for traditional CBT. In the sensitivity analysis, we found that our model was insensitive to most model inputs.

The case study analysis suggested that although mobile CBT is most effective and cost-saving when used only for treatment (i.e., used for individuals with moderate and severe GAD), it may also be beneficial when used for prevention only or both prevention and treatment. This suggests that offering mobile CBT to persons with mild anxiety (not clinically diagnosed GAD) who otherwise would not receive any preventive interventions could provide value to both society and payers. However, in a situation where the ability to deploy the mobile CBT program was limited, perhaps by short-term budget, the most impactful population to target would be moderate and severe GAD patients. This concept should be further developed once more robust efficacy data becomes available.

Online and mobile CBT programs may increase access to treatment for persons with GAD by making CBT more accessible and by removing some structural barriers (e.g., patients do not need to drive to their physician’s office for therapy). The availability of online CBT programs can then theoretically increase the proportion of persons with GAD who initiate therapy and reaps the downstream benefits of less anxiety and associated comorbidities. Although access to treatment is not only impacted by logistical and structural barriers, and our case study analysis does not explicitly quantify this increase in uptake, the comparison of the mobile CBT program and the status quo of no CBT can serve as a proxy for the potential QALYs gained and costs saved by expanded CBT access.

There were a few limitations to our proposed model and framework for analysis. We did not factor the effectiveness of pharmacotherapy in combination with CBT into transition rates due to a lack of GAD progression data specific to whether a person is on medication and how long they have been on medication. We also assumed that persons were on pharmacotherapy for their entire life. However if, as our case study suggested, mobile CBT’s clinical response rate is better than the clinical response rate of traditional CBT, we can assume that mobile CBT would result in more persons discontinuing GAD medications sooner. This could result in a greater reduction in overall costs compared to other strategies. Lastly, we made the conservative assumption that persons are only at increased risk for suicide once they are in one of the comorbidities states. If individuals in the GAD-only states also experience an increased risk of suicide, then even more QALYs would be gained and costs would be further reduced when mobile CBT is compared to traditional CBT and status quo, as shown in our case study sensitivity analysis.

There were several limitations of our case study analysis as well. First, the mobile CBT program effectiveness data came from a small pilot program of 89 individuals from one large national employer group. Therefore, the generalizability of the results is limited. However, results of sensitivity analysis suggest that mobile CBT may be cost-saving even if effectiveness is lower than in the pilot program. Secondly, the effectiveness data used for the traditional CBT intervention was based on an overall clinical response rate, and did not directly provide severity-specific effectiveness rates. The completion rates for mobile and traditional CBT’s effectiveness were also not directly comparable. To address these discrepancies, we varied the clinical response rate of traditional CBT in sensitivity analyses and found that only when traditional CBT’s clinical response rate increases to 76% does it become the preferred intervention over mobile CBT. We also made assumptions on the sustained effectiveness of mobile and traditional CBT. We assumed that both CBT interventions had the same sustained effectiveness, but results may change if there is a large discrepancy in effectiveness length for mobile CBT and traditional CBT. We also assumed that there are always some residual effects of the CBT programs throughout a person’s lifetime. As shown in our sensitivity analysis, if CBT’s effectiveness gradually decreases until it reaches the status quo transition rates in just 2 years, then mobile CBT is still cost-saving compared to traditional CBT and status quo.

The findings of the case study analysis, which are specific to a GAD population, are consistent with the results of other studies that examined the health outcomes and economic benefits of internet-based or mobile app versions of CBT programs for individuals with depressive and anxiety disorders [17, 41]. The estimated QALYs gained and costs saved by deploying this program as prevention, treatment, or both are meaningful. The case study results hold for both a societal perspective, taking into account disability days due to GAD, as well as for a more limited payer perspective, looking only at health-related costs. This suggests that payers, patients, employers and society overall could benefit from using internet-based mobile app CBT programs for GAD. This analysis should be rerun once more robust efficacy data with a larger sample size is made available for the mobile CBT program. In addition, further research on intervention-specific completion rates and long-term effectiveness in broader and more diverse GAD populations would allow for an even better understanding of the value of such programs.

## Supporting information

S1 Table Summary of transition probabilities and intervention effectiveness. (DOCX)

S2 Table Summary of utilization parameters for 3-month period. (DOCX)

S3 Table Reduction in overall costs breakdown, base case. (DOCX)
