# Supplementary material for: Mobile and traditional cognitive behavioral therapy programs for generalized anxiety disorder: A cost-effectiveness analysis
Source: PLoS One. 2018 Jan 4;13(1):e0190554. doi: 10.1371/journal.pone.0190554 (PMC5754075; doi:10.1371/journal.pone.0190554)
Supplement: S1 Table — (DOCX) [file pone.0190554.s001.docx]

**S1 Table.** Summary of Transition Probabilities and Intervention Effectiveness.

| **Parameter** | **Value** | **Source** |
| --- | --- | --- |
| **Disease Parameters** |  |  |
| 3-month transition probabilities, % |  |  |
| None-None | 92.6 | Calculated |
| None-Mild | 1.5 | [1] |
| None-Moderate | 1.5 | [1] |
| None-Severe | 4.4 | [1] |
| Mild-None | 6.9 | [2] |
| Mild-Mild | 89.4 | Calculated |
| Mild-Moderate | 1.9 | [3, 4] |
| Mild-Severe | 1.9 | [3, 4] |
| Moderate-None | 12.0 | [1] |
| Moderate-Mild | 20.0 | [1] |
| Moderate-Moderate | 57.1 | Calculated |
| Moderate-Severe | 6.6 | [1] |
| Moderate-Moderate with Comorbidities | 4.2 | [5] |
| Severe-None | 10.8 | Calculated [1] |
| Severe-Mild | 9.0 | Calculated [1] |
| Severe-Moderate | 9.0 | Calculated [1] |
| Severe-Severe | 67.0 | Calculated |
| Severe-Severe with Comorbidities | 4.2 | [5] |
| Moderate with Comorbidities-Moderate | 10.0 | [1, 6] |
| Moderate with Comorbidities-Severe with Comorbidities | 19.9 | [3, 6] |
| Moderate with Comorbidities-Moderate with Comorbidities | 70.09 | Calculated, assumed |
| Moderate with Comorbidities-Death due to Suicide | 0.01 | [7] |
| Severe with Comorbidities-Moderate | 10.0 | [1, 6] |
| Severe with Comorbidities-Severe with Comorbidities | 85.5 | Calculated, assumed |
| Severe with Comorbidities-Moderate with Comorbidities | 4.5 | [1, 6] |
| Severe with Comorbidities-Death due to Suicide | 0.02 | [7] |
| **Intervention Parameters** |  |  |
| Mobile CBT 3-month effectiveness, % |  |  |
| Mild-None | 38.0 | Lantern pilot study data |
| Mild-Mild | 57.0 | Lantern pilot study data |
| Mild-Moderate | 0.0 | Lantern pilot study data |
| Mild-Severe | 5.0 | Lantern pilot study data |
| Moderate-None | 29.0 | Lantern pilot study data |
| Moderate-Mild | 59.0 | Lantern pilot study data |
| Moderate-Moderate | 9.0 | Lantern pilot study data |
| Moderate-Severe | 3.0 | Lantern pilot study data |
| Severe-None | 23.0 | Lantern pilot study data |
| Severe-Mild | 50.0 | Lantern pilot study data |
| Severe-Moderate | 23.0 | Lantern pilot study data |
| Severe-Severe | 4.0 | Lantern pilot study data |
| Traditional CBT 3-month effectiveness, % |  |  |
| Mild-None | 42.0 | Calculated [8] |
| Mild-Mild | 55.7 | Calculated |
| Mild-Moderate | 1.2 | Calculated [8] |
| Mild-Severe | 1.2 | Calculated [8] |
| Moderate-None | 21.0 | Calculated [8] |
| Moderate-Mild | 21.0 | Calculated [8] |
| Moderate-Moderate | 49.0 | Calculated |
| Moderate-Severe | 6.0 | Calculated [8] |
| Severe-None | 14.0 | Calculated [8] |
| Severe-Mild | 14.0 | Calculated [8] |
| Severe-Moderate | 14.0 | Calculated [8] |
| Severe-Severe | 54.6 | Calculated |

CBT: cognitive behavioral therapy

**REFERENCES**

1. Rodriguez BF, Weisberg RB, ME P, Bruce SE, Spencer MA, Culpepper L, et al. Characteristics and predictors of full and partial recovery from generalized anxiety disorder in primary care patients. J Nerv Ment Dis. 2006;194(2):91-7. PubMed Central PMCID: PMC16477186

2. Ballenger JC, Davidson JRT, Lecrubier Y, Nutt DJ, Borkovec TD, Rickels K, et al. Consensus statement on generalized anxiety disorder from the International Consensus Group on Depression and Anxiety. J Clin Psych. 2001;62(Suppl 11):53-8.

3. Yonkers KA, Dyck IR, Warshaw M, Keller MB. Factors predicting the clinical course of generalised anxiety disorder. Br J Pyschiatry. 2000;176:544-9. PubMed Central PMCID: PMC10974960.

4. Karsten J, Hartman CA, Smit JH, Zitman FG, Beekman AT, Cuijpers P, et al. Psychiatric history and subthreshold symptoms as predictors of the occurrence of depressive or anxiety disorder within 2 years. Br J Pyschiatry. 2011;198(3):206-12. PubMed Central PMCID: PMC21357879

5. François C, Despiégel N, Maman K, Saragoussi D, Auquier P. Anxiety disorders, major depressive disorder and the dynamic relationship between these conditions: treatment patterns and cost analysis. J Med Econ 2010;13(1):99-109. PubMed Central PMCID: PMC20078336.

6. Bruce SE, Yonkers KA, Otto MW, Eisen JL, Weisberg RB, Pagano M, et al. Influence of psychiatric comorbidity on recovery and recurrence in generalized anxiety disorder, social phobia, and panic disorder: a 12-year prospective study. Am J Psych. 2005;162(6):1179-87. PubMed Central PMCID: PMC15930067.

7. Khan A, Khan SR, Leventhal RM, Brown WA. Symptom reduction and suicide risk in patients treated with placebo in antidepressant clinical trials: a replication analysis of the Food and Drug Administration Database. Int J Neuropsychopharmacol. 2001;4(2):113-8. PubMed Central PMCID: PMC11466159

8. Hunot V, Churchill R, Silva de Lima M, Teixeira V. Psychological therapies for generalised anxiety disorder. Cochrane Database of Syst Rev. 2007;(1):CD001848. PubMed Central PMCID: PMC17253466.
