# Supplementary material for: Mobile and traditional cognitive behavioral therapy programs for generalized anxiety disorder: A cost-effectiveness analysis
Source: PLoS One. 2018 Jan 4;13(1):e0190554. doi: 10.1371/journal.pone.0190554 (PMC5754075; doi:10.1371/journal.pone.0190554)
Supplement: S2 Table — (DOCX) [file pone.0190554.s002.docx]

**S2 Table.** Summary of Utilization Parameters for 3-Month Period.

| **Parameter** | **Value** | **Range** | **Source** |
| --- | --- | --- | --- |
| **Physician office visits** |  |  |  |
| No anxiety | 1.2 | 1.0 – 1.4 | [1] |
| Mild anxiety | 1.7 | 1.4 – 2.0 | [1] |
| Moderate anxiety | 2.2 | 1.8 – 2.6 | [1] |
| Severe anxiety | 2.4 | 1.9 – 2.9 | [1] |
| Moderate anxiety with comorbidities | 3.6 | 2.9 – 4.3 | [2] |
| Severe anxiety with comorbidities | 3.9 | 3.1 – 4.7 | [2] |
| **ER Visits** |  |  |  |
| No anxiety | 0.014 | 0.011 – 0.017 | [3] |
| Mild anxiety | 0.019 | 0.015 – 0.023 | [3] |
| Moderate anxiety | 0.025 | 0.020 – 0.030 | [3] |
| Severe anxiety | 0.027 | 0.022 – 0.032 | [3] |
| Moderate anxiety with comorbidities | 0.042 | 0.034 – 0.050 | [2, 3] |
| Severe anxiety with comorbidities | 0.046 | 0.037 – 0.055 | [2, 3] |
| **Hospitalizations** |  |  |  |
| No anxiety | 0.014 | 0.011 – 0.017 | [3] |
| Mild anxiety | 0.019 | 0.015 – 0.023 | [3] |
| Moderate anxiety | 0.025 | 0.020 – 0.030 | [3] |
| Severe anxiety | 0.027 | 0.022 – 0.032 | [3] |
| Moderate anxiety with comorbidities | 0.042 | 0.034 – 0.050 | [2, 3] |
| Severe anxiety with comorbidities | 0.046 | 0.037 – 0.055 | [2, 3] |
| **Disability days** |  |  |  |
| No anxiety | 3.9 | 3.1 – 4.7 | [1] |
| Mild anxiety | 7.5 | 6.0 – 9.0 | [1] |
| Moderate anxiety | 10.7 | 8.6 – 12.8 | [1] |
| Severe anxiety | 16.8 | 13.4 – 20.2 | [1] |
| Moderate anxiety with comorbidities | 16.6 | 13.3 – 19.9 | [4] |
| Severe anxiety with comorbidities | 28.1 | 22.5 – 33.7 | [4] |

ER: emergency room

**REFERENCES**

1. Spitzer RL, Kroenke K, Williams JB, Löwe B. A brief measure for assessing generalized anxiety disorder: the GAD-7. Arch Intern Med. 2006;166(10):1092-7. PubMed Central PMCID: PMC16717171.

2. Zhu B, Zhao Z, Ye W, Marciniak MD, Swindle R. The cost of comorbid depression and pain for individuals diagnosed with generalized anxiety disorder. J Nerv Ment Dis. 2009;197(2):136-9.

3. Berger A, Edelsberg J, Bollu V, Alvir JMJ, Dugar A, Joshi AV, et al. Healthcare utilization and costs in patients beginning pharmacotherapy for generalized anxiety disorder: a retrospective cohort study. BMC Psych. 2011;11(193). PubMed Central PMCID: PMC22151689

4. Kroenke K, Spitzer RL, Williams JB. The PHQ-9: validity of a brief depression severity measure. J Gen Intern Med. 2001;16(9):606-13. PubMed Central PMCID: PMC11556941.
