# Supplementary material for: Mobile and traditional cognitive behavioral therapy programs for generalized anxiety disorder: A cost-effectiveness analysis
Source: PLoS One. 2018 Jan 4;13(1):e0190554. doi: 10.1371/journal.pone.0190554 (PMC5754075; doi:10.1371/journal.pone.0190554)
Supplement: S3 Table — (DOCX) [file pone.0190554.s003.docx]

**S3 Table.** Reduction in Overall Costs Breakdown, Base Case.

| **Parameter** | **Mobile CBT Compared to Traditional CBT, million $^a^** | **Mobile CBT Compared to Status Quo, million $** **^a^** |
| --- | --- | --- |
| **Societal perspective** | 2,234 | 4,546 |
| Healthcare costs | 340 (15.2%) | 604 (13.3%) |
| Disability days | 1,894 (84.8%) | 3,941 (86.7%) |
| **Payer perspective** | 339 | 605 |
| Physician office visit | 146 (43.0%) | 327 (54.0%) |
| ER | 9 (2.7%) | 20 (3.2%) |
| Hospitalization | 139 (41.1%) | 280 (46.3%) |

CBT: cognitive behavioral therapy; ER: emergency room

**^a^** Costs are net present values (3% annual discount rate) over the cohort lifetime
